# Inflammation: improving understanding to prevent or ameliorate kidney diseases

**DOI:** 10.1590/1678-9199-JVATITD-2020-0162

**Published:** 2021-10-18

**Authors:** Sheila Marques Fernandes, Mirian Watanabe, Maria de Fátima Fernandes Vattimo

**Affiliations:** 1 Animal Model Experimental Laboratory (LEMA), School of Nursing (EEUSP), University of São Paulo (USP), São Paulo, SP, Brazil.; 2 Health Sciences and Wellbeing (CISBEM), University Center of United Metropolitan Colleges, São Paulo, SP, Brazil.

**Keywords:** Inflammation, Acute kidney injury, Kidney diseases, Biomarkers

## Abstract

Inflammatory processes are believed to play an important role in immune response to maintain tissue homeostasis by activating cellular signaling pathways and releasing inflammatory mediators in the injured tissue. Although acute inflammation can be considered protective, an uncontrolled inflammation may evolve to tissue damage, leading to chronic inflammatory diseases. Inflammation can be considered the major factor involved in the pathological progression of acute and chronic kidney diseases. Functional characteristics of this organ increase its vulnerability to developing various forms of injuries, including acute kidney injury (AKI) and chronic kidney disease (CKD). In view of translational research, several discoveries should be considered regarding the pathogenesis of the inflammatory process, which results in the validation of biomarkers for early detection of kidney diseases. Biomarkers enable the identification of proinflammatory mediators in kidney affections, based on laboratory research applied to clinical practice. Some inflammatory molecules can be useful biomarkers for the detection and diagnosis of kidney diseases, such as neutrophil gelatinase-associated lipocalin, kidney injury molecule-1 and interleukin 18.

## Background

Inflammatory processes are involved in the immune response, which maintains tissue homeostasis during adverse conditions, such as infections and other injuries. These processes result in the reduction of tissue function and act by enabling the healing process [1,2].

Pain, heat, redness and swelling are universal signs that accompany all inflammatory processes, which result from dilation of venules and arterioles, enhanced blood vessel permeability, blood flow with percolation of leukocytes into the tissues and release of inflammatory mediators [3].

Historically, four signs have been associated with wounds and infections for thousands of years, as described by the Roman doctor Cornelius Celsus in the 1^st^ century AD. In 1846 and 1867, Augustus Waller and Julius Cohnheim, respectively, described the main characteristic of an acute inflammatory process by leukocyte emigration from the blood vessels and vascular changes. Another important advance in this field was that of Elie Metchnikoff, in 1892, who discovered phagocytosis and the key role of macrophages and microphages (neutrophils) in host defense and in the maintenance of tissue homeostasis. This idea is the current concept that acute inflammatory response is a protective and survival tool of the tissue [1,4].

In this context, inflammation can be considered a major factor involved in the pathological progression of acute and chronic diseases in the kidney. Functional characteristics of this organ increase its vulnerability to developing various forms of kidney affections, such as acute kidney injury (AKI) and chronic kidney disease (CKD). Translational research in this area should consider several discoveries on the pathogenesis of inflammatory processes originated from laboratory-based research - the bench - bringing relevant findings that emerged from cell culture or animal model experiments (*in vitro* or *in vivo*). The response from the bench should be applied in the clinic practice - the bedside - in conducting both clinical research and clinical trials. Translational researches reinforce the strength of multidisciplinary science teams by emphasizing life-saving therapies in different diseases and evidence-based practice [5].

## Methods

This systematic review was developed following the guidelines of Proffered Reporting Items for Systematic Reviews and Meta-Analysis (PRISMA). The search was performed in the following electronic databases: PubMed, Google Scholar and SciELO. No due date was set for the publication. The descriptors were used according to Medical Subject Headings (MeSH) on PubMed, Google Scholar and SciELO: “acute kidney injury” *or* “kidney disease” *and* “inflammation”. The search was limited to English and Portuguese languages. 

The selection of the studies was performed in a standardized manner by two independent reviewers based on reading the title and abstract. Article analysis was performed when the title and abstract were not elucidative. A third reviewer ensured the specificity and quality of the process. Other information was obtained through online resources and the reference list of relevant cohort epidemiological, observational and experimental studies were consulted for inclusion in this review. 

## Results and Discussion

**Figure 1.**
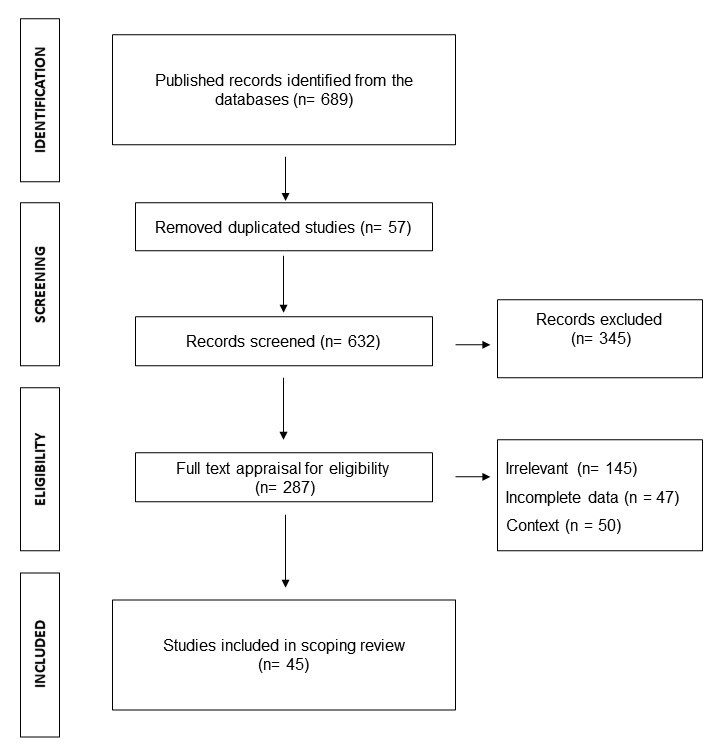
PRISMA flowchart shows the study design process.

Based on the selection process, 689 studies titles and abstracts were included in the identification process; 345 studies were excluded, 287 studies were selected for full text analysis and 45 studies were included in the final review. The PRISMA flowchart is shown in Figure 1. 

### Inflammatory response

Several factors, including a simple skin cut, infections, injury, cell damage and toxins can trigger a long molecular chain and cellular activity that are designed to eliminate potentially injurious stimuli. The inflammatory response follows four steps:

1. Injury or infection detected by receptors of the innate immune system [1,6].
2. Initiation of the inflammatory response in the body, involving non-infectious or infectious families of receptors expressed in tissue-resident macrophages, dendritic cells, and mast cells [7].
3. Production of inflammatory cytokines, chemokines, bioactive amines, eicosanoids and bradykinin that enhances blood vessel permeability induced by vasodilatation of venules and arterioles with migration of leukocytes into the tissues [3,8].
4. Infiltration of leukocytes, which play a major protective response in tissue injury by neutralizing and eliminating potentially injurious stimuli aiming to resolve the acute inflammatory response. Tissue progresses to resolution by recovery of the homeostasis and elimination of dead cells [9].

### Oxidative stress and inflammatory response

Inflammation processes and oxidative stress are pathophysiological events that are linked to each other. Under physiological circumstances, normal cellular metabolism produces reactive oxygen species (ROS) and reactive nitrogen species (RNS) that are efficiently eliminated in the presence of antioxidant defense mechanisms [10,11]. Thus, inflammatory cells generate a number of ROS and RNS that initiate intracellular signaling cascading and enhanceing proinflammatory gene expression [6,8]. 

Excessive production of ROS and RNS can modify the redox balance in favor of oxidative stress, and play a crucial role in tissue injury [10,11]. Oxidative stress is manifested by the increased production of signaling species, such as nitric oxide (NO) and hydrogen peroxide (H_2_O_2_), and strong oxidants, such as superoxide anions (O_2_^•-^) and hydroxyl radical (OH^•-^). Both ROS and RNS deplete and reduce antioxidant capacity, and can cause injury in all vital cellular components by oxidation of proteins, peroxidation of lipids, and DNA damage, leading to cell death [12]. Evidence from experimental data has demonstrated association of inflammatory response and oxidative stress in many diseases [13]. 

### Acute inflammation and chronic inflammation

The inflammatory process is a coordinated response by activation of cellular signaling pathways and release of inflammatory mediators that act in the injured tissue. Inflammation is a complex of physiological responses to a foreign organism, including human pathogens and a variety of particles and viruses. Inflammations are divided into acute and chronic inflammation, depending on various inflammatory processes and cellular mechanisms. It has been considered the major factor for the progression of various chronic diseases/disorders, including diabetes, cancer, cardiovascular diseases, kidney disease, eye disorders, arthritis, obesity, autoimmune diseases, and inflammatory bowel disease. Free radical production from biological and environmental sources and its imbalance with natural antioxidants further lead to several inflammatory associated diseases [6, 7]. 

Acute Inflammation is a short immune response and self-limited process, lasting from minutes to a few days. The active immune cells generate inflammatory mediators, enhanced microvascular permeability, leakage of plasma proteins or fluid and movement of leukocytes into the extravascular area, characterized by classic signs of heat, redness and swelling. In addition, inflammatory mediators increase sensitivity of the tissue to pain [9,14]. 

The main feature of inflammatory mediators is the short half-life when the injury stimulus is removed and thus acute inflammatory elements, in an active process, result in biosynthesis of active mediators - pro-resolving mediators - that act to promote tissue homeostasis [8,15,16,17]. The effects of prolonged inflammation response are associated with unresolved acute inflammatory phases and can be induced by persistent injury, infection or prolonged exposure to toxic agents, leading to a chronic inflammation, tissue destruction, fibrosis and necrosis. 

This chronic tissue inflammation has been associated with several consequences by biological response with enhanced risk of chronic diseases and disorders [18,19]. 

Acute inflammation is a defense mechanism to maintain health, characterized by a series of cellular and molecular events that reduce the progression of the injury or infection [1]. The acute inflammation is protective, but uncontrolled inflammation may lead to tissue damage, causing chronic inflammatory diseases. Currently, inflammation can be considered a major factor involved in the pathological progression of acute and chronic diseases in different organs, such as the heart, pancreas, liver, kidney, lung, brain, intestinal tract and reproductive system. This process depends on the nature of the etiology stimulus and location in the body [20]. 

Evidence for uncontrolled inflammation is an important component for many chronic diseases, including cardiovascular diseases, inflammatory bowel disease, neurodegenerative diseases such as Alzheimer’s and Parkinson’s, asthma, cancer, diabetes and autoimmune diseases [18,21].

### Kidney

Physiologically and metabolically, the kidneys are the most complex organs in the body. The kidney is a functional organ that is able to perform excretion of waste products from the metabolism, maintenance of water and electrolyte homeostasis, and endocrine functions. The functional units of the kidneys are the nephrons, consisting of the glomerulus (endothelial cells) and long tubule segments (epithelium cells). Kidneys are perfused with 20 to 25% of cardiac output to 125 mL/minute of filtrate to pass through to Bowman capsule that is reabsorbed through the peritubular capillary network and produces about 1.5 L of daily urine. These functional characteristics of the organ increase the vulnerability to developing various forms of kidney injury - AKI and CKD [22]. 

AKI is defined as an abrupt decrease in renal function, resulting from several insults in glomerulus - post-infectious glomerulonephritis, lupus nephritis, IgA glomerulonephritis - in tubule - renal ischemia (shock, surgery, hemorrhage, trauma, bacteremia, pancreatitis, pregnancy), nephrotoxic drugs (antibiotics, antineoplastic drugs, contrast media, organic solvents, anesthetic drugs, heavy metals) and endogenous toxins (myoglobin, hemoglobin, uric acid) [23]. The prevalence of AKI varies from 1 to 25% and mortality from 15 to 60%, and that reaches up to 50 to 60% in patients in intensive care units [23,24]. 

Inflammation response plays a major role in the pathophysiology of AKI. In the kidneys, the acute inflammatory response results in enhanced endothelial cell-leukocyte adhesion that compromises blood flow in renal microvasculature. Leukocytes can be activated by inflammatory mediators, such as cytokines, ROS, RNS and eicosanoids. In response to the inflammation, the renal tubule epithelium cells also generate mediators that potentiate the cascade of inflammation [25]. 

Experimental studies of AKI - ischemia and reperfusion model, sepsis-endotoxemia model, nephrotoxic model - have demonstrated leukocytes, including neutrophils, macrophages, natural killer cells, and lymphocytes infiltrating into the injured area, which resulted functional changes in vascular endothelial cells or in tubular epithelium cells [3,26]. 

Renal endothelial cells, tubular epithelium cells and inflammatory cells in complex cross-talk perpetuate up-regulated of cytokines and chemokines, ROS and RNS, and vasoconstrictors - prostaglandins, leukotrienes and thromboxane - which results in the infiltration of inflammatory cells into the kidney tissue. This mechanism may further determine the increase or decrease of the inflammation process in the kidney by generating pro-inflammatory or anti-inflammatory cytokines [3,26,27]. 

Chronic inflammation in tissue response by unresolved injury or infection is a biological response with several secondary consequences that enhance the risk of chronic diseases. Persistent inflammatory processes result in a negative effect on tissue function or in tissue damage. In addition, inflammatory cells contribute to dysregulated tissue repair response, accompanied by tissue remodeling, fibrosis, and persistent tissue metaplasia. The dysregulation of inflammation is similar to tissue pathology or decline on tissue function [1]. 

Molecular and cellular processes of chronic inflammation vary and depend on the type of inflamed cells and organs [28]. The progression of CKD is aggravated by chronic inflammatory responses. These processes result in glomerular inflammation, which causes proteinuria due to damage of the glomerular capillaries. Tubule injuries can cause fibrosis in kidney tissue [29,30]. In addition, oxidative stress by depletion of the endogenous intracellular antioxidant contributes to azotemia, high glomerular and tubular injury and elevated levels of inflammatory mediators. Both chronic inflammatory and oxidation of renal cells decrease the glomerular filtration, which contributes to progressive renal damage and dysfunction [31].

CKD is a global health problem with a prevalence of 5 to 10% of the world population and is associated with high morbidity and mortality. Progression of CKD results in end-stage kidney disease that requires kidney replacement therapy - dialysis or transplantation [32]. 

CKD is also described by decreased glomerular filtration rate (GFR) and cytokines elimination, enhancing levels of circulating cytokines and pro-oxidative metabolites. These consequences of CKD patients can be related to increased serum levels of nitrogenous wastes, metabolic acidosis and frequent infections [33]. 

Inflammation and immune dysregulation in CKD patients can be potentialized by hemodialysis treatment. The extracorporeal procedure results in upregulated inflammatory cytokines by impurities in dialysis water, microbiological quality of the dialysate, and bio incompatible factors in the extracorporeal dialysis circuit [34,35]. In addition, dialysis patients can be associated with frequent infectious and thrombotic events that contribute to inflammatory stimulations. These events include catheter-related bloodstream infections, access site infections, thrombosed intravascular fistulae and grafts, and episodes of peritonitis in peritoneal dialysis patients [36].

In summary, AKI predisposes to CKD and vice versa, and the inflammatory response plays a major role in the pathophysiology of both diseases.

*Kidney disease biomarkers*

In the view of translational research, several discoveries should be considered regarding the pathogenesis of inflammatory processes that result in the validation of biomarkers of kidney diseases from laboratory-based research - the bench. These discoveries have been applied in the clinic practice - the bedside - including biomarkers for the early detection of proinflammatory mediators.

The most recent diagnostic criteria is proposed by the Kidney Disease: Improving Global Outcomes (KDIGO) guidelines, which describe AKI based on changes in serum creatinine (SCr) and urine output (UO). AKI is defined by an increase in the SCr level to at least 0.3 mg/dL or more than 1.5 times the baseline within 48 hours, or a UO decrease to less than 0.5 mL/kg/h for 6 hours. KDIGO criteria stratifies three stages of AKI (Table 1) [37]. 

**Table 1.** Stages of acute kidney injury (AKI) [37].

Stage	SCr	UO
1	Increase in SCr ≥ 0.3 mg/dL (≥ 26.5 µmol/L) or increase in SCr ≥ 1.5 to 1.9 times	< 0.5 mL/kg/h (> 6 h)
2	Increase in SCr > 2.0 to 2.9 times	< 0.5 mL/kg/h (> 12 h)
3	Increase in SCr ≥ 3 times or increase in SCr to ≥ 4 mg/dL (≥ 353.6 µmol/L) or initiation of renal replacement therapy	< 0.3 mL/kg/h (24 h) or anuria (12 h)

SCr: serum creatinine; UO: urine output.

CKD is defined by GFR decreased to less than 60mL/min/1.73m² for more than three months or kidney damage by albuminuria more than three months. Although increasing urinary albumin excretion and SCR levels, along with diminished GFR, are also important markers of kidney function decline [38].

CKD is categorized into stages of severity to diagnosis and management of the disease (Table 2) [38].

**Table 2.** Stages of chronic kidney disease (CKD) [38].

Functional Criteria		Structural Criteria	
Stage	GFR	Stage	Albumin excretion rate to creatinine ratio
1	GFR > 90mL/min/1.73m² (normal)	A1	< 30 mg/g - normal to mildly increased
2	GFR 60 to 89 mL/min/1.73m²	A2	30 to 300 mg/g - moderately increased
3a	GFR 45 to 59 mL/min/1.73m²	A3	> 300 mg/g - severely increased
3b	GFR 30 to 44 mL/min/1.73m²		
4	GFR 15 to 29 mL/min/1.73m²		
5	GFR < 15 mL/min/1.73m²		

GFR: glomerular filtration rate.

Currently, the diagnostic criteria of kidney diseases are based on changes in both SCr and UO, where AKI is defined by increased SCr and decreased UO. CKD is detected by measures of kidney damage by albuminuria and decreased GFR [23]. Thus, SCr and UO are markers that have been applied in clinical practice. 

SCr has poor sensitivity in the setting of kidney injury. This marker is not ideal because it is affected by several factors such as age, gender, muscle mass, fluid administration and secretion of some drugs. On the other hand, UO is an early marker for renal dysfunction that can be affected by a patient's volemic and hemodynamic status or the administration of diuretics, so it is difficult to measure UO without a urinary catheter [39,40]. 

The ideal biomarker can predict and diagnose different types and etiology of kidney disease or the location of the injury. The use of non-invasive and easily accessed samples provides rapid results, predicts outcomes and enables the initiation and monitoring of therapeutic interventions. Various molecules have been identified in the systemic circulation and in glomerular filtration markers of glomerular function, such as enzymes that are released by tubular cells into the urine after tubular cell injury, described as markers of tubular damage, or inflammatory mediators released by renal cells or infiltrating inflammatory cells, described as markers of damage and indicators of site of injury [41,42]. 

The use of the concept “from the bench to bedside” to in vivo or in vitro studies have resulted in promising renal biomarkers from molecules that report glomerular filtration and tubule injury, and non-renal biomarkers derived from molecules that were filtered, secreted or reabsorbed, molecules that are constitutive or upregulated or molecules from immune cells infiltration [41]. Whereas some inflammatory molecules from basic science could be a useful biomarker for detection and diagnosis of kidney disease, the biomarkers that stand out in this context are the neutrophil gelatinase-associated lipocalin (NGAL), kidney injury molecule-1 (KIM-1) and interleukin 18 (IL-18).

*Neutrophil gelatinase-associated lipocalin*

Neutrophil gelatinase-associated lipocalin (NGAL) is a protein associated with gelatinase from neutrophils [43,44]. In physiological state, NGAL protein levels are very low in various biological fluids. It is excreted from the liver, spleen and kidneys [45]. NGAL is upregulated and released into the urine and plasma in the cellular injury. Because NGAL is a low molecular weight and positive charge that undergoes glomerular filtration, filtered NGAL is deleted by endocytosis from the apical membrane and it appeared in the urine [46].

Multicenter studies demonstrated that patients with elevated levels of NGAL without elevated SCr were at higher risk of death than patients with elevated SCr alone [47]. Early detection of NGAL has also been described that urinary NGAL accurately discriminated patients who developed AKI from patients that did not in hospitalized cirrhotic patients [48], and elevated serum NGAL levels were associated with the risk of developing AKI in acute decompensated heart failure patients [49]. 

*Kidney injury molecule-1*

Kidney injury molecule-1 (KIM-1) is produced by proximal tubular cells after ischemic or nephrotoxic injury, and its mRNA levels increase more than any other gene after kidney injury [50]. 

Extensive KIM-1 expression was found in proximal tubule epithelial cells in human kidney biopsy sections from patients with acute tubular necrosis [51]. Folic acid and cisplatin nephrotoxic models of AKI demonstrated that the upregulation of KIM-1 expression precedes the rise of SCr and serves as a general biomarker for tubular injury [52]. Additionally, urinary KIM-1 levels were significantly higher in patients with ischemic AKI compared to patients with other etiologies of AKI [53]. KIM-1 has been described as an important predictor of AKI in different scenarios of clinical practice - in post cardiac surgery patients and critically ill patients and, KIM-1 elevations occurred within hours of renal injury [54,55]. 

Initial clinical studies suggested that increased urinary KIM-1 level is a novel biomarker, and KIM-1 was approved by the United States Food and Drug Administration as a biomarker for clinical drug development [41]. 

*Interleukin 18*

Interleukin 18 (IL-18) is a member of the interleukin-1 cytokine superfamily and is known as an interferon-γ-inducing factor that regulates innate and adaptive immunity response [56]. This cytokine is produced by mononuclear cells, macrophages and non-immune cells including proximal tubule cells. IL-18 activity has been described in several inflammatory diseases, such as inflammatory arthritis, multiple sclerosis, inflammatory bowel disease, chronic hepatitis, systemic lupus erythematosus and psoriasis [56]. Upregulated expression of renal IL-18 has been shown in ischemia-reperfusion injury, inflammatory nephritis, and cisplatin-induced nephrotoxicity [57]. 

Urine IL-18 levels were increased in patients with acute tubular necrosis compared by prerenal azotemia, urinary tract infection, CKD and nephrotic syndrome [58]. and, in the higher levels of IL-18 has been shown to be an early marker of AKI and good predictor of mortality in critically ill patients [59]. Also, systemic review and meta-analysis studies described that urine IL-18 may be useful for predicting the development of AKI [60]. 

*Promising biomarkers of kidney diseases*

Several biomarkers have been applied to the early diagnosis of kidney disease in animal models or specific clinical settings, demonstrating the potential to improve patient care. Kidney tissues are heterogeneous, and currently the approaches focus on localizing specific portions of the nephron and representing distinct mechanisms of kidney disease responses in the process of kidney injury [61].

*IL-6*

IL-6 is a proinflammatory mediator. Acute renal insult involves inflammatory response, and IL-6 has been shown to be an inflammatory marker in renal patients, performing the systemic inflammatory marker C-reactive protein. Increased serum or urinary of IL-6 levels are an early marker of kidney disease [61].

*Soluble TNF receptors*

Soluble TNF receptors (TNFR1 and TNFR2) are circulating biomarkers of inflammation. TNF receptors play an important role in the progression of atherosclerotic and kidney disease and are associated with the progression of diabetic nephropathy to CKD stage 3 and end-stage renal disease [62].

*5-methoxytryptophan*

5-methoxytryptophan (5-MTP) is an endogenous tryptophan metabolite and is converted by tryptophan hydroxylase-1 enzyme. 5-MTP has anti-inflammatory activities. Chen et al demonstrated that tryptophan showed the same trend with 5-MTP in CKD patients, serum 5-MTP levels decreased with progression of CKD, and treatment of 5-MTH in unilateral ureteral obstruction animal models attenuated renal interstitial fibrosis by reducing inflammatory signaling molecules [63].

*Fatty acids*

Kidney function can affect the blood level and urinary excretion of metabolites. Therefore, changes in lipid metabolism can contribute to progression of CKD. Lipid disorders accelerated atherosclerosis and cardiovascular diseases in CKD patients [20, 33].

Feng and colleagues have described that fatty acid metabolism was associated with albuminuria change, decreasing polyunsaturated fatty acids - docosahexaenoic acid (DHA), docosatrienoic acid (DTA) and transient receptor potential (TTA) - and increasing saturated fatty acids (SFA), such as 5,8-tetradecadienoic acid (5,8-TDA) and eicosadienoic acid (EDA) in CKD patients with microalbuminuria and macroalbuminuria [64].

## Conclusions

Inflammation is a defensive response to infections or tissue injury and has the physiological purpose of restoring tissue homeostasis. However, an excessive inflammatory response is detrimental and induces negative effects on tissue function. The acute phase of the inflammatory response is a beneficial and self-limited process. Nevertheless, chronic phase can be associated with dysregulated repair with tissue remodeling, fibrosis, and persistent tissue metaplasia, which leads to the decline or complete loss of normal tissue function. Injured kidneys with morphological or functional changes in endothelial cells and in tubular epithelium cells induce the generation of inflammatory mediators that contribute to the evolution of several kidney diseases.

The understanding of inflammatory response pathways and the discovery and validation of biomarkers of kidney diseases in a variety of clinical settings contribute to the improvement in preventing and treating renal inflammatory diseases.

The use of translational strategies of research provides an opportunity to improve outcomes from scientific discoveries to developclinical practice by evidence-based interventions.

### Abbreviations

5-MTP: 5-methoxytryptophan; AKI: acute kidney injury; CKD: chronic kidney disease; GFR: glomerular filtration rate; H_2_O_2_: hydrogen peroxide; IL: interleukin; KIM-1: kidney injury molecule-1; NGAL: neutrophil gelatinase-associated lipocalin; NO: nitric oxide; O_2_^•-^: superoxide anions; OH^•-^: hydroxyl radical; RNS: reactive nitrogen species; ROS: reactive oxygen species; SCr: serum creatinine; UO: urine output.
